# Does activation of oxytocinergic reward circuits postpone the decline of the aging brain?

**DOI:** 10.3389/fpsyg.2023.1250745

**Published:** 2023-12-29

**Authors:** Benjamin Buemann

**Affiliations:** Retired, Veksø, Denmark

**Keywords:** brain, hypothalamus, inflammaging, learning, neuroplastic, oxytocin, reward, sexual

## Abstract

Oxytocin supports reproduction by promoting sexual- and nursing behavior. Moreover, it stimulates reproductive organs by different avenues. Oxytocin is released to the blood from terminals of oxytocinergic neurons which project from the hypothalamus to the pituitary gland. Concomitantly, the dendrites of these neurons discharge oxytocin into neighboring areas of the hypothalamus. At this location it affects other neuroendocrine systems by autocrine and paracrine mechanisms. Moreover, sensory processing, affective functions, and reward circuits are influenced by oxytocinergic neurons that reach different sites in the brain. In addition to its facilitating impact on various aspects of reproduction, oxytocin is revealed to possess significant anti-inflammatory, restoring, and tranquilizing properties. This has been demonstrated both in many *in-vivo* and *in-vitro* studies. The oxytocin system may therefore have the capacity to alleviate detrimental physiological- and mental stress reactions. Thus, high levels of endogenous oxytocin may counteract inadequate inflammation and malfunctioning of neurons and supportive cells in the brain. A persistent low-grade inflammation increasing with age—referred to as inflammaging—may lead to a cognitive decline but may also predispose to neurodegenerative diseases such as Alzheimer’s and Parkinson. Interestingly, animal studies indicate that age-related destructive processes in the body can be postponed by techniques that preserve immune- and stem cell functions in the hypothalamus. It is argued in this article that sexual activity—by its stimulating impact on the oxytocinergic activity in many regions of the brain—has the capacity to delay the onset of age-related cerebral decay. This may also postpone frailty and age-associated diseases in the body. Finally, oxytocin possesses neuroplastic properties that may be applied to expand sexual reward. The release of oxytocin may therefore be further potentiated by learning processes that involves oxytocin itself. It may therefore be profitable to raise the consciousness about the potential health benefits of sexual activity particularly among the seniors.

## Introduction

An intertwined interplay between oxidative stress and a chronic low-grade inflammation, referred to as inflammaging, appears to be essential for the loss of resilience of the aging organism. An increased formation of Reactive Oxygen Species (ROS) results in enhanced inflammatory activity which, in turn, may further promote ROS production (Zuo et al., 2019). Dysfunctional mitochondria due to accumulating mutations in their DNA may be an important initiating and promoting factor for the overproduction of ROS with age (Chen G. et al., 2020). The resulting oxidative stress which can damage DNA and protein structures of various cell systems seems to be a general factor for several age-associated pathologies. In the brain, inflammaging may not only lead to different conditions of dementia but also involve the gradual decline in the regulatory capacity of neuroendocrine systems. In addition, the brain releases different tissue protective and reparative substances both internally and to the general circulation. This function may be impaired by a deterioration of the cells that produce the substances. Initiatives that counteract inflammation in the brain may therefore be an interesting possibility to extend health span. This may particularly apply to the hypothalamus as the superior defender of body homeostasis. The present article argues that cerebral inflammaging can be counteracted by behavioral stimulation of reward mechanisms involving oxytocin.

## Chronic micro-inflammation accentuated by stress responses accelerates neurodegenerative brain conditions

Inflammaging is accepted as an underlying condition that predisposes to degenerative diseases. This includes type-2 diabetes, cardiovascular disease, dementia, and some cancer diseases (Rea et al., 2018). It has been proposed that accumulation of cells which have entered a senescent state is a major cause of inflammaging (Cevenini et al., 2013). Senescent cells are characterized by an enhanced release of pro-inflammatory cytokines and a loss of proliferative capacity. This also applies to the brain where the microglia are believed to contribute to the inflammaging and senescence processes (Angelova and Brown, 2019). Microglia constitutes a type of immune cells that resides in the brain and other parts of the central nervous system. Microglia is important for the brains immune defense but it is also crucial as support for the nerve cell function. In elderly individuals, the microglia become more susceptible to acquire an inflammatory phenotype when stimulated by circulating pro-inflammatory cytokines (Perry and Teeling, 2013). The aged brain therefore becomes more prone to inflammatory nerve damages as a result of systemic inflammations. In this context, inflammaging in different parts of the brain appear to be important for the development of neurodegenerative conditions that, in turn, may lead to depression or dementia including Alzheimer’s disease (Santos et al., 2016).

However, age-associated inflammatory mechanisms may also be involved in non-pathological alterations of the aging brain. Pro-inflammatory cytokines may impair synaptic long-term potentiation (LTP) when their activity is elevated. The natural gradual decline of learning capabilities in the seniors may therefore be associated with inflammaging. Particularly, brain-derived neurotrophic factor (BDNF) signaling may be disrupted (Patterson, 2015). BDNF is essential for both structural and functional synaptic plasticity. In particular, cognitive and affective functions have been suggested to be affected by a reduced development of neural progenitor cells into mature neurons in the hippocampus (Chesnokova et al., 2016).

The intrinsic age-associated alterations in the brain might be accelerated by chronic mental stress. The biological processes observed with this condition resemble the deterioration observed with aging to some extent. In this case, an increased inflammatory level caused by an inappropriate activation of the microglia can also occur (Niraula et al., 2017). The age associated cell senescence that impairs neurogenesis and neuroplasticity may therefore be aggravated by mental stress responses. In this context, a small hippocampal volume and a poor memory performance in elderly subjects have been found to be associated with a high and increasing level of plasma cortisol over the years (Lupien et al., 1998). Stress which leads to excessive activation of the hypothalamic–pituitary–adrenal (HPA) axis can therefore contribute to an elevated inflammatory state. In this situation, an augmented microglia response may be caused both by elevated levels in the brain of corticotropic releasing factor (CRF; Kritas et al., 2014) and by glucocorticoids entering the brain (Frank et al., 2012). Most population surveys find increased levels of plasma cortisol and a trend of a flattened diurnal cortisol curve in older individuals. At the same time, both measures are predictors of a negative health outcome particularly in seniors. However, positive social interactions and a robust stress coping capacity both appear to be positive health indicators and are accompanied by an improved cortisol regulation (Gaffey et al., 2016). Preventing a detrimental HPA axis activity could therefore be an approach to delay degenerative processes in the brain and other organs.

## Inflammation in the brain speeds up the aging of the body

Maintaining the different brain functions is significant for cognition and behavior. However, it is also essential for the health of the entire body. Thus, systemic homeostasis is defended by the hypothalamus and neural stem cells residing in this area appears to be important to preserve this function. The overall decline in body functions with age may therefore relate to a gradual decay of hypothalamic stem cells. In this context, accelerated aging processes were reported in mice having their hypothalamic stem cells selectively destroyed. This was demonstrated by a faster decline in muscle and mental functions. Moreover, the animals had a shorter lifespan (Zhang et al., 2017). In contrast, if the hypothalamic stem cells were renewed in mid-aged mice by stem cells from the hypothalamus of new-born mice the animals were aging at a lower rate. However, this effect was only seen if the implanted stem cells were manipulated to withstand the inflammatory conditions that prevails in the old mice. The aggravating of inflammatory activity in the hypothalamus may involve a naturally increasing activity of NF-κB (Zhang et al., 2013). NF-κB is a transcription factor that upregulates the expression of several pro-inflammatory genes. Accordingly, the aging could be accelerated or retarded in the mice if the NF-κB activity was, respectively, accelerated or delayed in the hypothalamus (Zhang et al., 2013). There is evidence that hypothalamic inflammaging is an important part of the pathology of degenerative maladies including type-2 diabetes (Tang et al., 2015). The decline with age in homeostatic capacity of different neuroendocrine cell systems in the hypothalamus may be caused by oxidative stress accompanied by an impaired neural stem cell function. Thus, an inappropriate inflammatory activity in the paraventricular nucleus (PVN) of the hypothalamus that leads to an increased production of ROS may cause sympathetic hyperactivity and hypertension (Cruz et al., 2015). Health- and lifespan might therefore be extended by factors that upregulate the activity of neural stem cells and downregulate inflammation in the hypothalamus.

## Oxytocin facilitates reproduction but does also support tissue maintenance

Oxytocin is released to the blood from the posterior pituitary gland. As a hormone it stimulates contractions of the myometrium (Blanks and Thornton, 2003), myoepithelium of the milk ducts (Crowley, 2015) and the epididymal tubules (Gupta et al., 2008). Its discharge is triggered by action potentials in oxytocinergic magnocellular neurons projecting from the hypothalamus (Wakerley and Lincoln, 1973). However, oxytocin also enhances social and sexual behavior via neurons projecting from the hypothalamus to different parts of the brain including the limbic system, prefrontal cortex, and brain stem (Jurek and Neumann, 2018). Finally, the dorsal horn of the spinal cord is also innervated by oxytocinergic neurons. Here oxytocin can moderate incoming pain signals (Condés-Lara et al., 2006) and facilitate genital reflexes (Oti et al., 2021). A promoting role of oxytocin in reproductive behavior has been traced back to the nematodes as the mating script in these worms is disturbed when the signaling of nematocin is ablated (Garrison et al., 2012). Nematocin is the homolog of oxytocin in nematodes.

Only one genuine oxytocin receptor has been described and it belongs to the G Protein-Coupled Receptor (GPCR) superfamily. The action of oxytocin is depending on the type of G-protein (Gq or Gi) that is activated. A great variety of intracellular responses can be elicited as many different signal transduction cascades can be affected. This has been outlined in Chatterjee et al. (2016). The behavioral effects of oxytocin including its anxiolytic effects appears to be dependent of the activation of both G-protein subtypes upstream to the stimulation of the Extracellular signal-Regulated protein Kinase (ERK) 1/2 cascade (Busnelli and Chini, 2018). In addition to its promotion of reproduction, protective and restorative processes are also stimulated by oxytocin. In this regard, oxytocin can activate the Nitric Oxide Synthetase (NOS) enzymes (eNOS and nNOS) which may dampen cellular oxidative stress (Gonzalez-Reyes et al., 2015) by the activation of a Gq-protein.

Oxytocin also affects the metabolism and growth of the cells. Its activation of Gq-protein increases the intracellular Ca^2+^ level which may stimulate the AMP activated protein kinase (AMPK) pathway (Lee et al., 2008; Florian et al., 2010). AMPK stimulates autophagy and pathways that enhance the energy efficiency of the cells. Furthermore, Mammalian target of rapamycin (mTOR) which promotes protein synthesis may be downregulated by oxytocin (Klein et al., 2013). Oxytocin therefore seems to have the capacity to put cells into a light catabolic state Reproduction enhances the risk for organisms to pass into an energy deficit. In this situation, the release of oxytocin may constitute a feed-forward mechanism to increase the chance of survival by improving energy management. Apart from ensuring the energy supply of cells during shortage of fuel, autophagy is important for the clarence of defective cell components including ineffective mitochondria (Li and Chen, 2019). In the brain, an impaired autophagy may cause the accumulation of amyloid β protein in Alzheimer’s disease (Perluigi et al., 2015). Moreover, oxytocin has been proposed to contribute to restoration of injured liver tissue as it was found to promote autophagy in isolated hepatocytes (Luo et al., 2021).

During severe cellular stress, oxytocin may promote signaling pathways that protect against cell death (Polshekan et al., 2019). Moreover, different types of stem cells may be stimulated to differentiate and proliferate via an enhancement of ERK1/2 pathways and it is therefore probable, that oxytocin has both protective, restorative, and anti-senescence properties (Noiseux et al., 2012; Elabd et al., 2014; Cho et al., 2019; Ge et al., 2019). This opens up for a possible application of oxytocin to treat different atrophic conditions. Such a potential has already been demonstrated in different settings. Histological appearance and function of the vaginal mucosa in post-menopausal women has been reported to improve after topical treatment with oxytocin (Al-Saqi et al., 2015). Animal experiments have demonstrated that oxytocin can have preserving and reparative effects on skeletal muscles (Elabd et al., 2014), heart (Matsuura et al., 2004), structure and strength of bones (Elabd et al., 2008; Ge et al., 2019), intestinal mucosa (Chen et al., 2015), and skin lesions (Xu et al., 2017).

With regard to the enhancing effects of oxytocin on stem cells, mesenchymal stem cells preconditioned by oxytocin was found to improve their ability to survive and proliferate. Such treatment reinforced their potential to protect stressed cardiomyocytes from apoptosis (Noiseux et al., 2012) and to repair the damages after a myocardial infarct (Kim et al., 2012). The differentiation of muscle stem cell and their capacity to support repairment of skeletal muscles in old mice have also be shown to be enhanced by treatment with oxytocin (Elabd et al., 2014). It was therefore suggested that sarcopenia partly may be attributable to a declining impact of oxytocin with aging. In addition to improve stem cell function, oxytocin may also have anti-senescent effects on other cell systems. Thus, non-senescent fibroblast from young donors can convert into a senescent phenotype if they are exposed to a medium that is pre-conditioned by senescent fibroblasts. However, when oxytocin was added to the preconditioned medium, the young fibroblasts displayed a decreased senescence rate. This suggests that oxytocin may have a protecting impact against the transformation of non-senescent cells into senescence when exposed to signaling from senescent cells in their neighborhood (Cho et al., 2019).

## Oxytocin reduces inflammation and stress reactions in the brain

An anti-inflammatory effect of oxytocin may contribute to its tissue protecting capabilities. A role of oxytocin to alleviate harmful inflammation has been demonstrated in various of experimental models (Buemann et al., 2020). This has also been confirmed in a human study where lipopolysaccharide (LPS) bacterial toxin was infused to introduce systemic inflammation (Clodi et al., 2008). A downregulation of NF-κB signaling appears to be a factor in this effect of oxytocin (Tang et al., 2019). Inhibition of NF-κB causes the release of pro-inflammatory cytokines to be reduced. Moreover, macrophages may be more inclined to attain a restorative rather than inflammatory state. Furthermore, the potential of oxytocin to stimulate nuclear factor erythroid 2-related factor 2 (Nrf2) observed in human fibroblast (Cho et al., 2019) might also apply to nerve cells or other brain tissue cells. If so, this would be an additional pathway that oxytocin may counteract inflammatory damages. Nrf2 is a transcription factor that targets the antioxidative response element (ARE) and hereby promotes the transcription of an array of genes related anti-oxidative pathways in the cells. Nrf2 has been suggested to play a crucial neuroprotective role (Hannan et al., 2020). A protective role of oxytocin on the brains anti-oxidative defense systems is supported by a study where rats were treated with the mitochondrial toxin 3-NP. In this situation, intracerebroventricular injection of oxytocin improved the capacity of the ROS-degrading enzymes Superoxide Dismutase (SOD) and Catalase (CAT) in different brain regions (Moslemi et al., 2019).

That oxytocin has an anti-inflammatory effect in the brain has been documented by a mouse experiment. In this study, LPS was injected intraperitoneally which resulted in an inflammatory response in the prefrontal cortex. This inflammation could be reduced by nasally administration of oxytocin (Yuan et al., 2016). Accordingly, the response to LPS of isolated microglia can be attenuated by the addition of oxytocin to the medium (Yuan et al., 2016; Inoue et al., 2019). Thus, the increment of pro-inflammatory cytokines was abolished, and the morphologic alterations of the cells were reduced. The detection of oxytocin receptor expression on the microglia cells (Yuan et al., 2016) corresponds to these observations.

Oxytocin may attenuate chronic stress signaling in the brain and reduce its damaging consequences. An elevated CRF response to emotional stress has been reported in mice with ablated oxytocin production (Nomura et al., 2003). Endogenous oxytocin may therefore blunt the reactivity of the HPA axis. Oxytocin released from the dendrites of oxytocinergic neurons may dampen the activity of neighboring CRF-producing cells (Dabrowska et al., 2011). Moreover, other brain structures that are engaged in fear processing may communicate with oxytocinergic fibers projecting from the hypothalamus. This may establish a feedback system that moderates neuroendocrine stress responses (Windle et al., 2004; Cohen et al., 2010). In addition, oxytocin may protect hippocampal neurons against apoptosis during high levels of glucocorticoids by a receptor dependent mechanism (Latt et al., 2018). Mental stress may also trigger inflammatory stress reactions in the brain. Depressive-like symptoms can be observed in mouse pups after maternal separation which are accompanied by an increase in oxidative- and inflammatory stress markers in the hippocampus. However, these responses could be attenuated by intracerebroventricular injection of oxytocin (Amini-Khoei et al., 2017).

Brain-Derived Neurotrophic Factor (BDNF) is important for the formation of new neurons. It is also pivotal for the plasticity of synaptic structures engaged in learning processes (Cunha et al., 2010). Furthermore, BDNF protects nerve cells against oxidative stress (Yang et al., 2014). In this context, oxytocin may promote BDNF expression during sustained stressful conditions and therefore defend cognitive functions in the case of chronic stressful conditions (Dayi et al., 2015). Moreover, oxytocin may induce hippocampal nerve growth even when the level of glucocorticoids is elevated (Leuner et al., 2012). A neurophysiological study on slices from hippocampus achieved from rats supports that oxytocin can protect neural function against stress (Lee et al., 2015). It was shown that the impairment of the synaptic plasticity after the animals have been exposed to uncontrollable stress was reduced by antecedent treatment with oxytocin.

## Treatment with oxytocin counteracts the development of neurodegenerative diseases in animal *in-vivo* and *ex-vivo* models

Several studies in rodents have demonstrated the capacity of oxytocin to alleviate the damaging effects of a variety of neurotoxins when they are applied as models for different neurodegenerative conditions. This has been demonstrated with rotenone injected into substantia nigra pars compacta (SNc; Erbaş et al., 2012) and systemic treatment with MPTP (Almansoub et al., 2020) as models for Parkinson’s disease. Oral treatment with aluminum chloride has been used in rats as a model for Alzheimer’s disease as it induces a similar histopathology with accumulation of β-amyloid and Tau proteins. In that model, intranasal administration of oxytocin restored cognitive functions and diminished such lesions in the hippocampus (El-Ganainy et al., 2022). Synaptic plasticity can be directly impaired in slices dissected from hippocampus if treated with fragments of β-amyloid but can be reestablished if oxytocin is added to the media (Takahashi et al., 2020). Oxytocin has also been tested on the APP/PS1 transgenic mice which express abnormal amyloid precursor proteins that causes an early-onset Alzheimer’s disease. The disease is generally accompanied by an elevated microglia activity and inflammatory level in the brain. When the APP/PS1 mice were treated nasally with oxytocin microglia activity was attenuated and the β-amyloid aggregated in a less detrimental manner. Moreover, the impaired memory that characterizes the mice was improved by oxytocin (Selles et al., 2023). Oxytocin has the capacity to reduce the expression of Toll-like receptor 4 on microglia cell surface and—by so—inhibit the activation of pro-inflammatory signaling cascades in the cells. In a recent study, this was applied to construct anti-inflammatory nanoparticles loaded with oxytocin that can be released slowly to target. When the particles were tested by nasal administration to APP/PS1 Alzheimer mice their cognitive functions improved together with a diminished hippocampal atrophy and a preserved synaptic plasticity (Cheng et al., 2023).

Part of the neuroprotective effect of oxytocin may be mediated by activation of GABAergic pathways. Apart from constituting the major inhibitory system of the brain, GABAergic activity can play an important anti-inflammatory and neuroplastic role also in relation to trauma (Michalettos and Ruscher, 2022). Moreover, an impaired GABA-signaling in the hippocampus and prefrontal cortex has been proposed to contribute to the general decline in cognitive functions with age (McQuail et al., 2015). In this context, there is evidence that oxytocinergic stimulation of GABAergic interneurons in the hippocampus improves cognitive functions by increasing the signal-to-noise ratio when information is processed in the local nerve circuits (Owen et al., 2013).

## Oxytocin released to the circulation is coupled to a widespread oxytocinergic brain activity

When oxytocin is released to the general circulation from the pituitary gland it is triggered by activity in the magnocellular oxytocinergic neurons causing them to discharge oxytocin from their terminals. However, this neural activity may also result in dendritic release of oxytocin into the surrounding extracellular space in the hypothalamus. Here, the oxytocin may have some autocrine and paracrine effects. The autocrine effect may coordinate the burst of oxytocin discharge during labor and lactation (Ludwig and Stern, 2015). The paracrine signaling of oxytocin can affect other hypothalamic neurons, including the inhibition of the CRF-producing cells (Dabrowska et al., 2011). The latter mechanisms may facilitate lactation by exerting a tranquilizing impact. Vasopressin which has a molecular structure that is similar to oxytocin is also released from the dendrites of magnocellular neurons in the hypothalamus. Vasopressin has been found to diffuse over a distance up to 100 μm where it is able to exert paracrine effects (Son et al., 2013). Furthermore, oxytocin may travel within the hypothalamus and to more remote brain areas via the cerebrospinal fluid (CSF). Thus, oxytocin has been proposed to affect behavior by such a mechanism alternatively to signals carried by neurons (Veening et al., 2010). Widespread areas of the brain may therefore be affected by oxytocin produced in the hypothalamus. It has been argued that during adequate natural stimulation oxytocin may attain concentration in the range of 1 nM, in the hypothalamus and certain other brain areas which should be sufficient to induce cellular responses considering the receptor binding affinity of oxytocin (Busnelli and Chini, 2018). In this regard, similar and lower concentrations of oxytocin have been reported to elicit anti-inflammatory responses in different types of isolated immune cells (Buemann et al., 2020). Apart from its neuroendocrine functions, endogenous oxytocin may therefore have a role to protect and maintain brain tissue, particularly in the hypothalamus. A preserved high oxytocin activity in the seniors may therefore counteract degenerative processes in the brain. Preliminary data from a study in elderly humans support this notion (Ebner et al., 2019). It was reported that 4-week nasal administration of oxytocin had an increasing effect on amygdala, hippocampus, and putamen gray matter volume.

## Oxytocin promotes reproductive behavior by acting on different reward mechanisms

Oxytocin is an important facilitating factor for most aspects of reproductive behavior. However, the majority of our knowledge on this topic comes from rat experiments where the different cerebral oxytocin signaling systems have been stimulated or ablated—the latter by antagonists or by genetic engineering (Melis and Argiolas, 2021). It has been found that penile erection can be induced by injecting oxytocin in various of brain regions including Ventral Tegmental Area (VTA), amygdala, hippocampus and bed nucleus of stria terminalis but also nuclei in the hypothalamus itself, in particular, the paraventricular nucleus (PVN; Melis and Argiolas, 2021). It was suggested that sexual stimuli triggers input to the hypothalamus from the cerebral cortex. This, in turn, activates oxytocinergic nerves projecting from the PVN to various parts of the brain. Subsequently, when stimulated by oxytocin the different brain areas will feed back to the PVN by nerves releasing dopamine or other transmitters. This may constitute self-reinforcing mechanisms that finally triggers activity in neural pathways projecting from the PVN to the lower part of the spinal cord where they synapse with the genital nerves (Melis and Argiolas, 2021).

This complicated system of neural circuits does not appear to be limited to the promotion of penile erection but is probably applied for most sexual responses. Part of it constitute reward circuits by activating the mesolimbic pathway and therefore integrates sexual responses with reward. The mesolimbic system comprises dopaminergic nerves that project from the VTA to different subcortical hedonic hotspots including Nucleus Accumbens (NAc). However, such neurons also communicate with cortical regions involved in learning and reward (Alcaro et al., 2007). One demonstration of the dual function of these dopaminergic pathways is that injection of dopamine at the site of the oxytocinergic nerve somas in the hypothalamus both results in penile erection and an increased concentration of dopamine in the NAc (Succu et al., 2007). Moreover, oxytocinergic projections to different hedonic hotspots can modulate behavior. This occurs by an alteration of the reward values of various stimuli (Xiao et al., 2017) This is mediated by acting on several other signaling systems involved in reward including endocannabinoid activity in NAc (Wei et al., 2015). Oxytocin also appears to have the capacity generally to enhance the efficacy of opioid signaling (Meguro et al., 2018).

The orbitofrontal cortex is greatly involved in the perception and evaluation of rewarding stimuli as well as satiation in primates including humans. This requires integration of a rewarding stimulus with other potentially rewarding stimuli and with information about the internal state of the body. The engagement of the orbitofrontal cortex in the assessment of pleasure related to food consumption has been outlined in a recent article (Rolls, 2021). However, the orbitofrontal cortex appears to be essential for the overall estimation of pleasure (Berridge and Kringelbach, 2015). This also pertains to the pleasure of sex (Georgiadis and Kringelbach, 2012).

The oxytocin reaction to pleasant tactile skin stimulation may be primarily dependent on signal transduction via particular slow-conducting neurons (Löken et al., 2009; Walker et al., 2017). Interestingly, oxytocin, *per se,* appears to amplify the response to such input. This was indicated by an experiment where the arm of male subjects was stroked with different items (Chen Y. et al., 2020). When the subjects were treated nasally with oxytocin, they had a greater response both when tested objectively by fMRI assessment of the orbitofrontal cortex and when the men rated their perception of pleasantness. Oxytocin appears particular to reinforce the sexual quality of pleasurable touch. This was indicated by another experiment where heterosexual men were informed whether it was a man or a woman who stroked their calf (Scheele et al., 2014). It was found that treatment with oxytocin solely enhanced the perception of pleasure if the subjects believed that the strokes were performed by a woman. In accordance with this observation, oxytocin had a greater impact on the response of the orbitofrontal cortex if the men were convinced that they were experiencing a female touch. The reinforcement of the pleasure of touch may also apply to endogenous oxytocin. This is supported by the observation that a 50% increase in plasma concentration of oxytocin with foot massage was accompanied by an increased orbitofrontal activity (Li et al., 2019). A modest response in blood concentrations of oxytocin may be a result of an increase in the activity of the oxytocinergic neurons in the hypothalamus which is much more pronounced. This can be suggested based on a study, where rats were gently stroked on their back (Okabe et al., 2015).

## Sexual activity results in a significant oxytocin response

Gratifying social connections may have a promoting effect on the basal level of oxytocin activity (Tops et al., 2007). However, sexual activity appears to be one of the most effective triggers of oxytocin release together with childbirth and breastfeeding. Plasma oxytocin concentration has been found in most studies to increase both in men and women during sexual activity but to a various degree (Cera et al., 2021). However, these studies are mostly based on masturbation sessions in a very un-naturalistic setting and may therefore not be a reliable model for most sexual engagements which might trigger greater oxytocin responses. One of the experiments found more than a 4-fold increase in plasma oxytocin at ejaculation in men (Murphy et al., 1987). With regard to CSF, a 3-fold increase in oxytocin was found in cisterna magna 20 min after ejaculation in male rats (Hughes et al., 1987) whereas no elevation could be detected in lumbar CSF after orgasm in men and women (Krüger et al., 2006). The latter result may be explained by the sampling site. Hypersexuality has been reported to be associated with persistent higher blood levels of oxytocin in men (Flanagan et al., 2022).

The increase in blood and CSF oxytocin concentrations probably reflects an activation of neurons involved in the sexual response circuits. As mentioned afore, oxytocin may both be discharged in different regions of the brain and to the blood at the same time during activation of the oxytocinergic neurons in the PVN. The same neurons that have terminals which release oxytocin to the circulation from the pituitary gland may, in fact, have collateral axons which project to nuclei that are engaged in the reward system (Althammer and Grinevich, 2017). Both the duration and intensity of a sexual experience seem to determine how much oxytocin that is released. Plasma oxytocin has been found to increase during masturbation in both genders until it peaked at orgasm. However, in the women that were capable to achieve another orgasm, it continued to increase (Carmichael et al., 1987). Another experiment showed that the proportional rise from baseline to orgasm in plasma oxytocin concentration was positively correlated to pelvic muscle contraction during orgasm both in male- and female subjects (Carmichael et al., 1994). Furthermore, the proportional plasma oxytocin increase correlated with the intensity of their orgasmic experience in the multiorgasmic women (Carmichael et al., 1994). The opioid antagonist naloxone has been reported to blunt the perception of pleasure during the orgasm. At the same time, the response in plasma oxytocin was reduced. However, the increase in the heart rate and blood pressure at orgasm was unaffected by naloxone (Murphy et al., 1990). These observations suggest that the nerve activity experienced as sexual pleasure is integrated in the oxytocinergic nerve activity that is responsible for the release of oxytocin during sex. According to this notion, the reaction in serum oxytocin during coitus appears to be absent in anorgasmic women (Caruso et al., 2018).

Oxytocin may interact with testosterone to reinforce sexual behavior. The enzyme aromatase that is involved in the conversion of androgen to estrogen may be pivotal for the capability of testosterone to promote libido (Brooks et al., 2020). In the oxytocinergic neurons, aromatase is expressed predominantly in the neurons that communicate with the limbic system (El-Emam Dief et al., 2013). This suggests that the impact of oxytocin that may enhance the urge for sex takes place downstream of testosterone. Interestingly, oxytocin may promote testosterone release (Gossen et al., 2012). The Leydig cells may be directly stimulated by oxytocin to produce testosterone (Frayne and Nicholson, 1995). This might postpone the decline with age in blood testosterone that otherwise could result in a reduced libido (Allan et al., 2008).

Experiments have been conducted where oxytocin has been given nasally in the hope of enhancing sexual desire and pleasure. These studies have provided inconclusive results (Muin et al., 2015; Kruger et al., 2018) but they may show that the impact of such approach may be most significant in men (Behnia et al., 2014). The discouraging result of these investigations could be due to a relatively low entrance of oxytocin from the nasal cavity into the brain (Striepens et al., 2013) which is difficult to control. Similarly, the outcome of various clinical tests in which oxytocin were administered nasally for the treatment of different social deficits have also been ambiguous (Erdozain and Peñagarikano, 2020). Furthermore, the curve representing the dose–response relationship between the amount of intranasal oxytocin treatment and brain reactions may, in some cases, display an inverted U-shape rather than a steadily increase (Martins et al., 2022.). In general, it must be pointed out that the complicated network of oxytocinergic neurons that constitutes the substrate for the reward functions of oxytocin cannot be properly replicated by simply inject it through the nose. Negative outcomes of such investigations do therefore not invalidate the presence of a significant role of endogenous oxytocin in relation to the topic being tested.

## Oxytocin may enhance associative learning and modulate perceptive processes

Associative learning is important for sexual behavior. In this context, tactile and non-tactile sexual stimulation can act as unconditioned stimuli that may prompt a conditioned response. In one experiment, women had their clitoris stimulated at the same time as neutral pictures were shown repeatedly. A vaginal blood flow response could thereafter be observed if the same picture was displayed in absence of sexual stimulation whereas no such reaction was seen if the women were exposed to another unconditioned neutral picture (Both et al., 2008). It is disputed if classic conditioning can result in sexual fetishism (O'Donohue and Plaud, 1994). However, it is well-acknowledged that reproductive behavior can be altered substantially by an imprinting that is generated by different sexual experiences in humans as well as other mammals. Here, oxytocin may promote learning processes associated with reproductive and social behavior. A study on mating female mice has confirmed this contention (Fang et al., 2008). It was revealed that unimpaired oxytocin signaling was essential to form a memory of the partner’s scent that occurs during the mating. Moreover, it was shown the imprinting role of oxytocin was mediated by an amplification of the synaptic transmission (long-term potentiation, LTP) in the olfactory bulb (Fang et al., 2008). The pregnancies introduced by subsequent mice can be aborted if the scent of the first mouse is imprinted in the female. Thus, basal mechanisms including enhancement of LTP and neural plasticity may be in play when learning is supported by oxytocin (Pekarek et al., 2020). This may also apply to the improvement of social cognition and spatial orientation that may occur with birth and lactation (Monks et al., 2003). A general capability of oxytocin to support conditional learning has been disclosed in an unconventional setting (Eckstein et al., 2016). In that experiment healthy men were exposed to electric shocks while they were shown pictures of houses or faces. It was reported, that when the pictures were subsequently displayed the subjects had an increased fear response and activity of fear processing brain areas if they had been treated with oxytocin. Oxytocin also modulates behavior by influencing the processing of different inputs to the sensory cortex. This has been demonstrated in mice, which are responsive to the calls of their pups (Marlin et al., 2015). Here, the mothers’ auditory neurons are triggered by the particular sounds from their offspring. This response was demonstrated to be potentiated if ascending oxytocinergic neurons in the hypothalamus were stimulated. Moreover, the activity of inhibitory interneurons that sharpen the signal to noise ratio in the information processing may be enhanced by oxytocin (Owen et al., 2013). This may mean that the salience of pertinent sensory inputs might be increased. It was proposed that social cognition may be improved by this mechanism.

## Neuroplastic properties of oxytocin as a resource to enhance sexual reward?

It is plausible that oxytocin also facilitates reproductive behavior by improving the attentiveness and associative learning that occurs during sexual activity. By so, it may enhance sexual pleasure and motivation. The intensity of the sexual awareness may therefore be increased by approaches that amplify the oxytocin response. In other words, neuroplastic modulations being facilitated by oxytocin may be promoted in this way. This may mean that persistent improvements of the subsequent sexual experiences are established. Such a central mechanism may be similar to the development of chronic pain precipitated by maladaptive neuroplastic alterations in the sensory and cingulate cortices (Thibault et al., 2014) but it occurs in a more beneficial framing.

The self-reinforcing properties of the oxytocinergic system might therefore be applied in the context of sexual reward to potentiate the protective effects of oxytocin. This notion should be acknowledged when engaging in sexual activities. In this context, nipple stimulation may be a resource as it has been reported to increase sexual arousal in 51% and 78% young men and women, respectively (Levin and Meston, 2006). Furthermore, an agent which stimulates the arrector pili muscles in the nipples to contract has recently been found to enhance the intensity and pleasure of orgasms in women (Krychman et al., 2020). The intensifying effect of nipple stimulation on sexual pleasure may involve an amplified oxytocinergic response. Thus, an oxytocin reaction can be elicited by breast stimulation also in women who do not lactate (Chiodera et al., 1991). The presence of such oxytocin response in men does not seem to be elucidated. One study applying fMRI has reported that nipple stimulation activates the genital area of the sensory cortex (Komisaruk et al., 2011). This emphasizes the presence of a neural mechanism that may act synergistically with genital stimulation to enhance the activity of the sexual reward circuits and therefore the oxytocin release. In this situation, associative learning processes being facilitated by an elevated oxytocinergic activity may strengthen an intrinsic link between nipple stimulation and sexual reward.

## Can age-associated decay be delayed by sexual activity?

One intriguing study on middle-aged rats demonstrated that persistent sexual activity increased hippocampal neurogenesis to a level similar to that of young rats. Moreover, their cognitive functions and novelty seeking behavior were improved compared to a control group with no access to receptive females (Glasper and Gould, 2013). The involvement of oxytocin with its neurotropic properties is obvious, however, unfortunately no group with ablated oxytocin signaling to investigate such role was included in the study. Another interesting consequence of sexual activity on brain function is that it may not only promotes oxytocin release but also the expression of oxytocin receptors in the brain as reported in the hypothalamus of rats (Gil et al., 2013). This might counteract a possible decline with age in the expression of oxytocin receptors.

The putative systemic health benefits of sexual activity in humans are difficult to evaluate scientifically. However, there exists some observational investigations that support several health benefits of such activity. Thus, both a reduced all-cause mortality and a risk of cardiovascular disease have been found to be related to sexual activity (Palmore, 1982; Davey Smith et al., 1997; Cao et al., 2020). However, the most convincing support of a possible anti-aging effect of sexual activity may come from a cross-sectional study in women (Cabeza de Baca et al., 2017). The study tested the statistically determining factors for the length of the telomeres in blood cells. Different health indices, perceived stress, and quality of partner relationship were entered the multivariate analysis, however, recent sexual intimacy was the only variable that showed a highly significant association with telomere length. A study in rats indicates the presence of a preserving effect of oxytocin on telomeres (Faraji et al., 2018). In that study, social housing protected their telomeres against abbreviation, but this effect was ablated when an oxytocin antagonist was given. This advantages impact of oxytocin may be explained by a reducing effect on physiological stress. Telomeres are very vulnerable to oxidative stress (Reichert and Stier, 2017) and the activating effect of oxytocin on anti-oxidative mechanisms may play a role (Reichert and Stier, 2017).

Breast-feeding and sexual activity share many neurophysiological mechanisms including the activation of the hypothalamic oxytocin release. Several human studies indicate that breast feeding, *per se*, reduces the mother’s risk to experience cardiovascular diseases later in life (Buemann and Uvnäs-Moberg, 2020). A direct protective effect of circulating oxytocin may play a role. However, the autonomic nervous system may also be remodeled, possibly under the influence of oxytocin, in a way that is advantageous for metabolism, blood pressure, and the immune system.

The responsiveness of the oxytocinergic system at old age in humans may be important to address but the literature is sparse on that topic. However, in one recent study, the expression of the oxytocin receptors was reported to be upregulated in several brain regions in seniors compared to younger adults both in men and women (Rokicki et al., 2022). A few human studies have been conducted to elucidate whether treatment with oxytocin or oxytocin analogs may alleviate cognitive disability in elderly subjects with or without dementia (Barraza et al., 2013; Tampi et al., 2017; Grainger et al., 2018; Valdes-Hernandez et al., 2021). These studies have been focused on indicators of social cognition and wellbeing. The studies are based on relative short-term interventions and are inconclusive. They do not address the possible impact of a chronic enhancement of the oxytocinergic activity in the hypothalamus. In one study in elderly men, a negative association was actually found between plasma oxytocin and both crystallized and fluid cognitive skills (Polk et al., 1858). However, a systematic meta-analysis has been conducted on studies where central and peripheral oxytocin had been measured coordinately (Valstad et al., 2017). It was concluded that a significant correlation between the two measurements was only seen in studies where oxytocin had been administered nasally or in a setting where stress had been introduced experimentally whereas no correlation was observed if the samples had been taken at basal conditions. The application of oxytocin measured in the blood as a general proxy of central oxytocin levels can therefore be disputed.

## Conclusion

A combination of a sustained relatively high oxytocin activity established by a good social network and the oxytocin bursts elicited by sexual activity may be optimal. Sexual activity should therefore be accepted as an approach to health similar to physical training and dietary recommendations. This notion may particularly apply to the elderly individuals that may use to refer to sex as a vanishing remembrance. Younger individuals possibly having a greater neuroplasticity should embrace the notion that persistent imprinting of their sensory processing and reward circuits may be possible. In particular, this may enhance the reward of sexual activity and oxytocin responses which might increase their chance of a healthy old age.

### Comment

The present article is an expansion of a work previously being published by the same author (Buemann, 2022). It now provides further recent experimental evidence for a protective capacity of oxytocin against neurodegenerative conditions. Moreover, the present work further argues for the involvement of oxytocin in sexual reward mechanisms and for the integrity of the brain that this may have.

## Author contributions

The author confirms being the sole contributor of this work and has approved it for publication.
